# Appendiceal abscess complicated by mechanical small-bowel obstruction: a diagnostic and therapeutic challenge: A case report

**DOI:** 10.1097/MD.0000000000048190

**Published:** 2026-04-10

**Authors:** Kun Yang, Zhaopu Li, Yang Li, Jiaqing Lin, Tao Xu, Wei Zhu, Guangwei Gong

**Affiliations:** a Department of General Surgery, Xiaogan Hospital Affiliated to Wuhan University of Science and Technology, Xiaogan, Hubei Province, China; b Medical College, Wuhan University of Science and Technology, Wuhan, Hubei Province, China.

**Keywords:** appendiceal abscess, mechanical small-bowel obstruction

## Abstract

**Rationale:**

Appendiceal abscesses are typically managed conservatively. Mechanical small-bowel obstruction (MSBO) is a rare complication that poses diagnostic and therapeutic dilemmas.

**Patient concerns:**

A 52-year-old woman with appendiceal abscess, which also manifested as symptoms of intestinal obstruction, was finally confirmed to have MSBO intraoperatively.

**Diagnoses:**

Appendiceal abscess, MSBO.

**Interventions:**

The patient presented with 5-day migratory right lower quadrant pain. Initial computed tomography suggested appendicitis with possible small-bowel obstruction. Despite the use of aggressive antibiotics and electrolyte correction, the patient developed vomiting, obstruction, and worsening abdominal distension. A repeat computed tomography scan (day 6) confirmed appendiceal abscess with MSBO. Emergency laparoscopy was converted to laparotomy, which revealed a phlegmonous mass compressing the terminal ileum. Intraoperative frozen sections were used to exclude malignancy, and ileocecal resection with ileo-ascending colonic anastomosis was performed. The patient recovered uneventfully after the procedure.

**Outcomes:**

The patient resumed bowel function on postoperative day 3 and was discharged on postoperative day 9. At the 6-month follow-up, the patient did not experience any discomfort.

**Lessons:**

MSBO should be suspected when obstruction persists despite appropriate conservative treatment. An appendiceal abscess combined with MSBO requires early surgical intervention when conservative therapy fails. Timely imaging reassessment and multidisciplinary decision-making are critical to avoid delayed management.

## 1. Introduction

Appendiceal phlegmon and abscess account for 2% to 10% of acute appendicitis and are usually managed nonoperatively with antibiotics or percutaneous drainage.^[1]^ Mechanical small-bowel obstruction (MSBO) is not a direct complication of appendiceal abscess and is often misdiagnosed as a paralytic ileus or inflammatory obstruction, leading to delayed surgery. We present a case of appendiceal abscess-induced MSBO that resolved only after ileocecal resection, highlighting the key decision-making challenges.

## 2. Case presentation

### 2.1. History and examination

**Figure 1.**
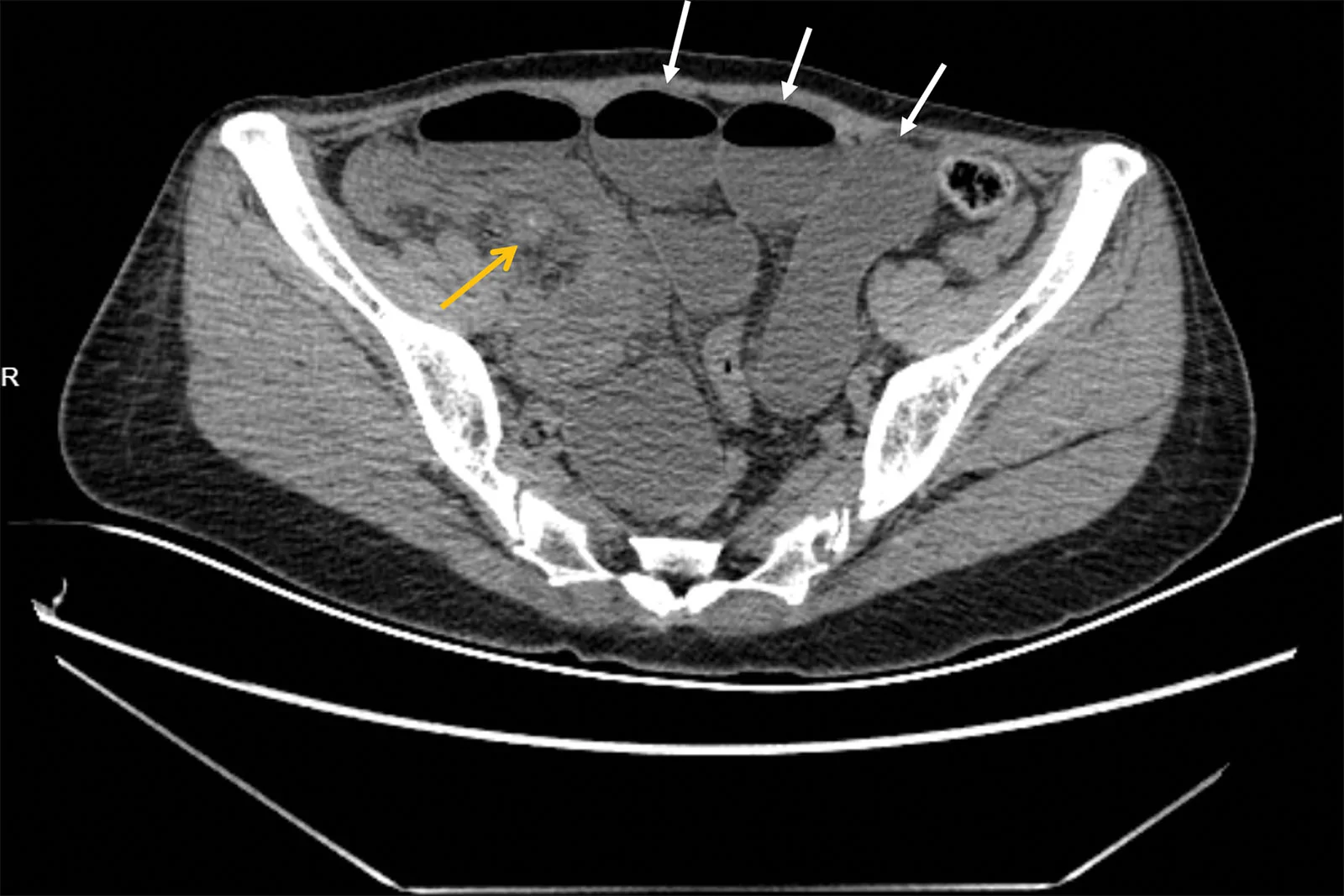
Thickening of the appendix (yellow arrow) and significant dilation of the small intestine (white arrow).

A 52-year-old woman presented with 5-day migratory abdominal pain localized to the right lower quadrant (RLQ). She has no history of abdominal surgery, no history of RLQ inflammatory episodes, no history of small-intestinal obstruction, and no history of inflammatory bowel disease, tuberculosis, trauma, or any condition capable of causing adhesions. She underwent 3 days of antibiotic treatment before admission. Her vital signs were stable (T: 37.3°C). Abdominal examination revealed RLQ tenderness without rebound. Initial laboratory tests revealed hypokalemia (K^+^): 3.43 mmol/L (reference range: 3.5–5.5 mmol/L), neutrophilic increase: white blood cell 6.98 × 10^9^/L (reference range: 3.5–9.5 × 10^9^/L), N% 79.7%, and elevated C-reactive protein 103.94 mg/L (reference range < 5 mg/L). Computed tomography (CT) suggested appendiceal inflammation with small-bowel dilation (Fig. 1).

### 2.2. Clinical course

**Figure 2.**
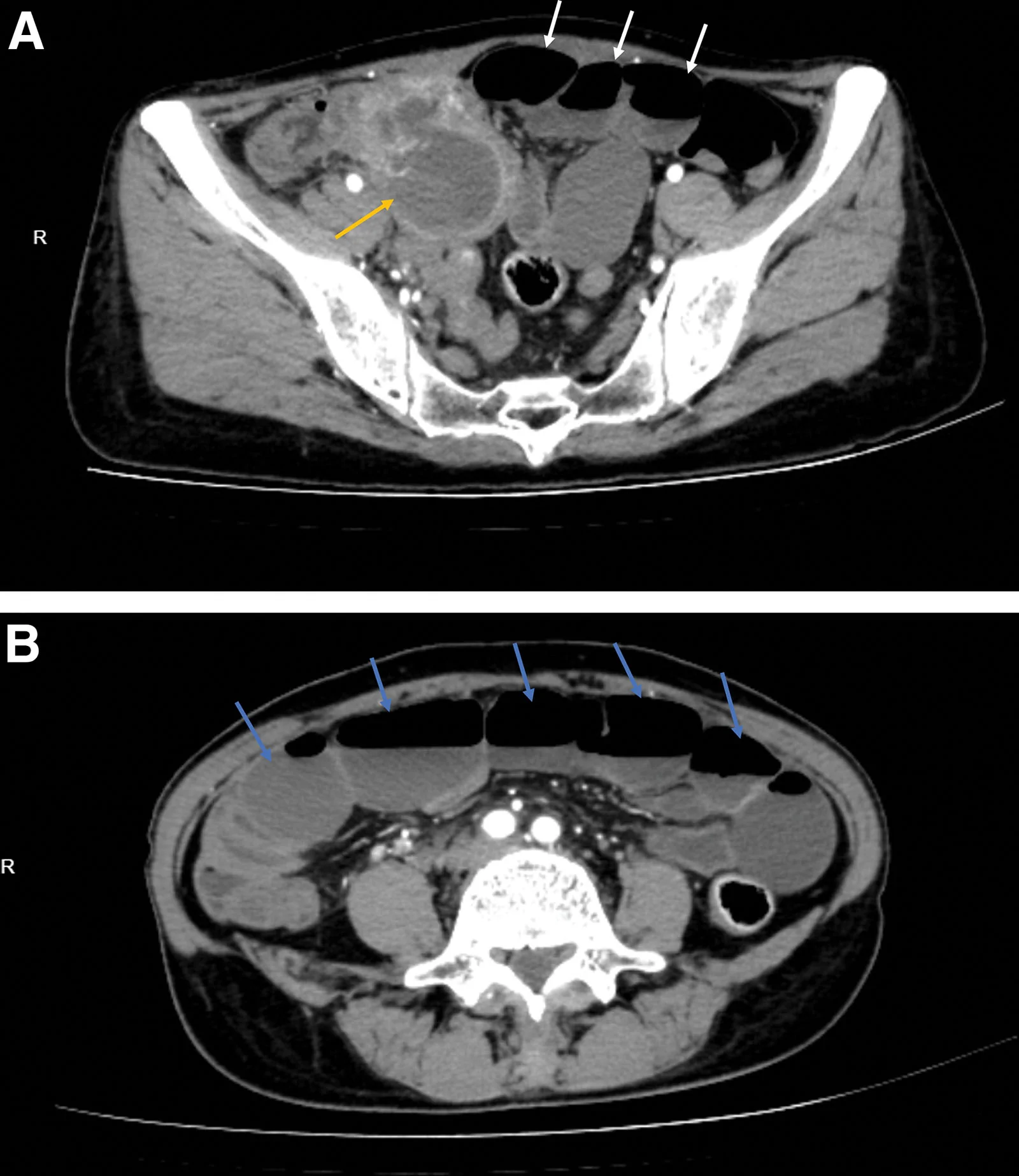
(A) Contrast-enhanced CT image showing a well-defined appendiceal abscess (yellow arrow) causing upstream small-bowel dilation (white arrow). (B) The small-bowel dilation did not show significant relief (blue arrow). CT = computed tomography.

Despite antibiotics (cefoperazone-sulbactam plus metronidazole) and potassium correction, she developed vomiting and obstipation on day 4. We had adopted standard nonsurgical treatment measures, including fasting, nasogastric, and fluid resuscitation, but there was no effect. Repeat CT (day 6) was conducted, and 2 radiology specialists were involved in reassessment. They pointed out that there was an appendiceal abscess (5 × 4 cm) and small-bowel dilation in the CT image, and the tumor cannot be ruled out in the ileocecum (Fig. 2). Labs indicated worsening leukocytosis (white blood cell: 16.13 × 10^9^/L, N%: 88.4%) and persistent inflammation (C-reactive protein: 129.02 mg/L).

### 2.3. Surgical intervention

**Figure 3.**
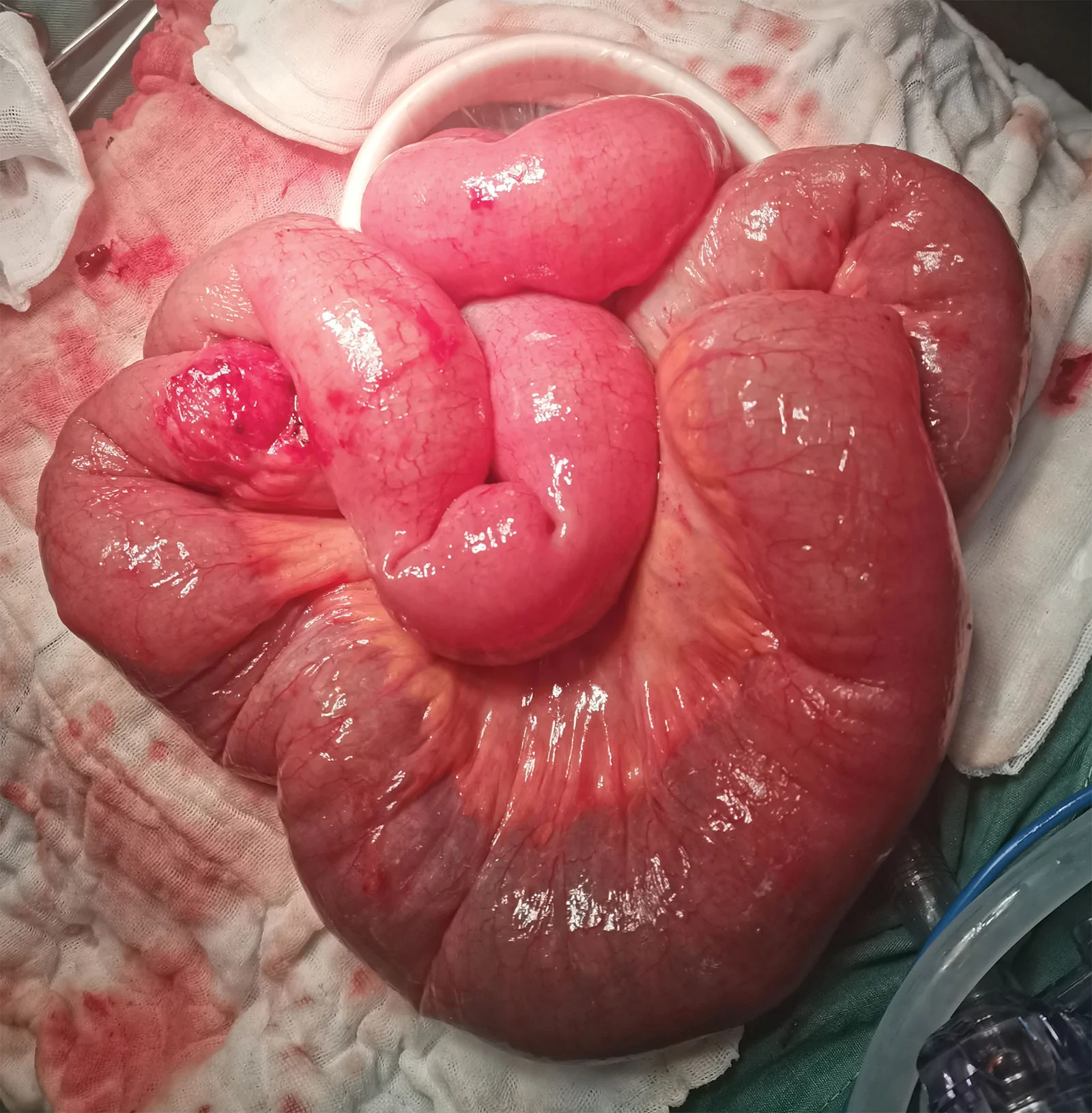
Laparotomy image of small intestine dilation; the obstruction point is located at the terminal ileum.

**Figure 4.**
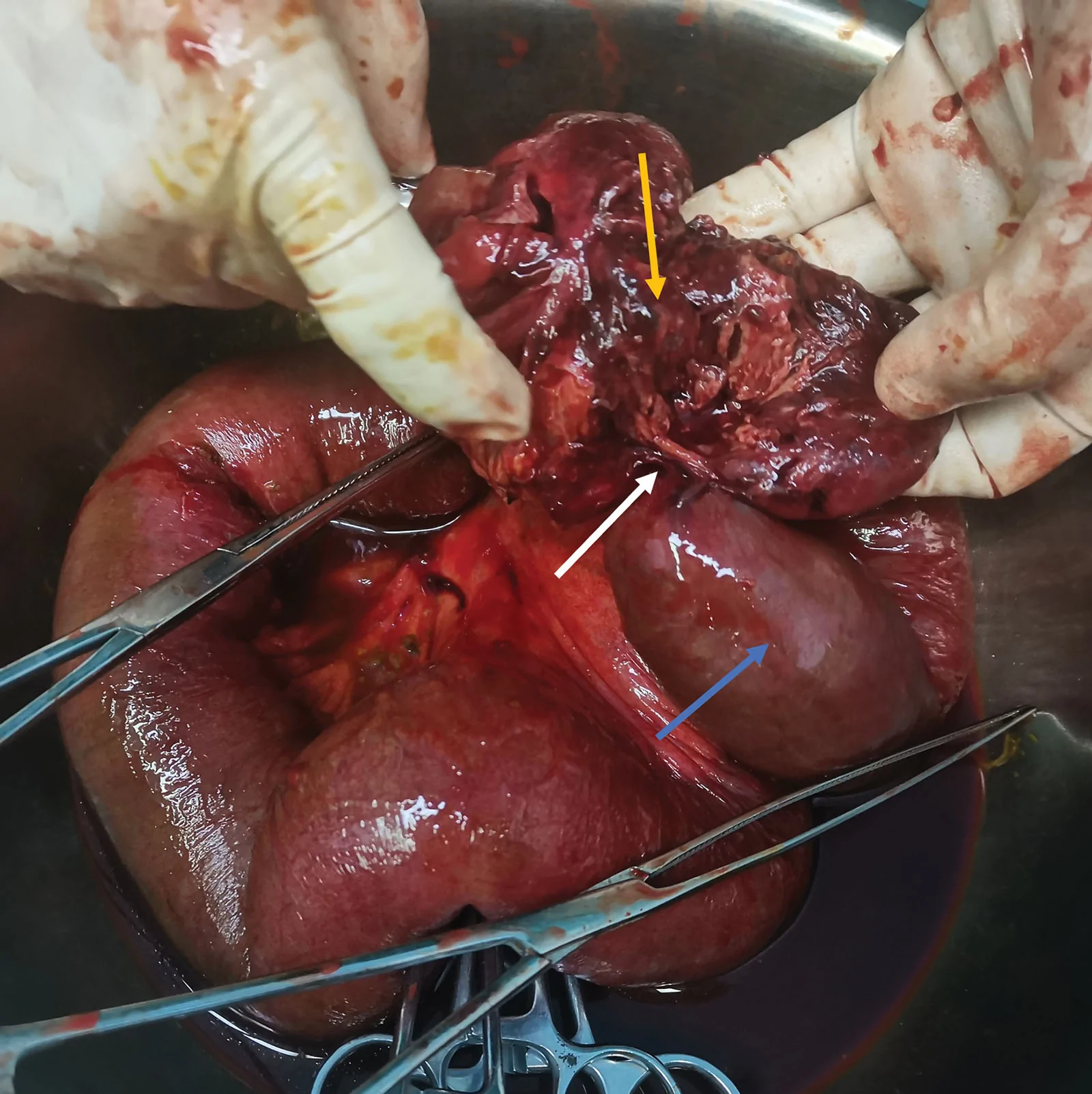
Postoperative specimen: fibrotic band formation (white arrow), small intestine (blue arrow), ileocecal region (yellow arrow).

The patient’s symptoms persisted without relief, and there were obvious manifestations, including abdominal pain, bloating, vomiting, and peritonitis. The bowel sounds are active, and the patient’s mental state is poor, indicating MSBO and indications for surgical exploration. For this reason, we had detailed communication with the patient’s family and explained the necessity of surgical exploration and possible situations that may occur during the operation. The patient’s family expressed great understanding and willingness to accept the surgery. Emergency laparoscopy (day 8) revealed a mass in the ileocecum. We can also see obvious dilation of the small intestine and enhanced intestinal peristalsis. The transition point is located at the end of the ileum. Partial adhesion between the small intestine and colon (location of appendiceal abscess) did not cause obstruction (Supplemental video). Conversion to laparotomy revealed a terminal ileal stricture and significant dilation of the small intestine (Fig. 3). There was an abscess in the right lower abdomen, and the appendix had been perforated. Intraoperative biopsy and rapid frozen pathology excluded malignancies (only inflammatory changes). Ileocecal resection with side-to-side ileo-ascending anastomosis was performed. In the postoperative specimen, we found that there was a cord (fibrotic band formation) at the end of the ileum that was strangling the small intestine (Fig. 4).

### 2.4. Outcome

The patient resumed bowel function on postoperative day (POD) 3, tolerated liquids on POD 5, and was discharged on POD 9. Histopathological examination confirmed perforated appendicitis with abscess formation. The patient’s postoperative recovery was well, with no abdominal pain. Routine hematological tests and a CT scan were normal. At the 6-month follow-up, the patient did not experience any discomfort.

**Figure 5.**
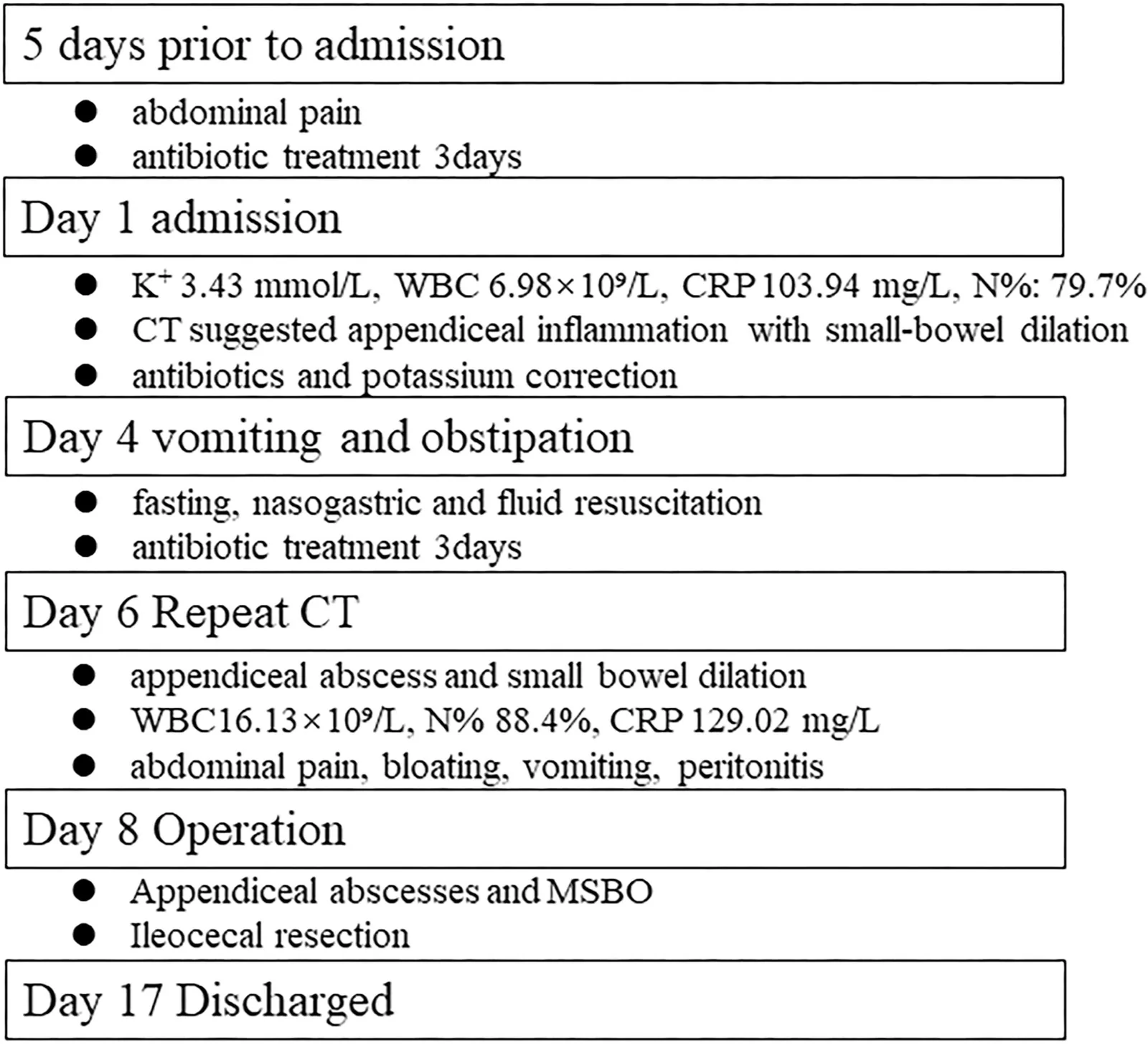
Historical and current information from this episode of care is organized as a timeline. CRP = C-reactive protein, CT = computed tomography, MSBO = mechanical small-bowel obstruction, WBC = white blood cell.

This paper presents the development of events using a timeline (Fig. 5) to enhance the reader’s understanding of their chronological sequence and interrelationships.

## 3. Discussion

An appendiceal abscess is a common surgical disease, often caused by untreated acute appendicitis.^[2]^ Inflammation of appendiceal abscess is mostly confined to the ileocecal area, and conservative treatment is often used to promote inflammation absorption, which generally causes less intestinal obstruction.^[3]^ This article reports a case of appendiceal abscess complicated by intestinal obstruction, where conservative treatment was ineffective, and recovery was achieved after surgical treatment.

For the treatment of adult appendiceal abscesses, the current view of most experts is conservative treatment, and if necessary, puncture drainage.^[4,5]^ The diagnosis of appendiceal abscess was confirmed upon admission based on the patient’s medical history, physical signs, and imaging examinations. Therefore, we actively treated the patient with antibiotics after admission. However, the patient experienced symptoms of intestinal obstruction after admission, with CT showing significant dilation of the small intestine. At this time, we faced diagnostic challenges regarding the etiology and treatment of intestinal obstruction. First, was the cause of intestinal obstruction in the patient mechanical or inflammatory? Appendicitis caused by suppuration or perforation, leading to abdominal infection and peritonitis, is very common, resulting in intestinal obstruction caused by small-intestinal inflammation. However, in this case, inflammation was relatively limited, and there were no obvious symptoms of peritonitis. Despite anti-infectious therapy, the obstruction persisted, suggesting a mechanical cause. Second, the patient had low potassium levels upon admission, which can also cause intestinal paralysis and intestinal obstruction.^[6]^ However, after active anti-infection and potassium supplementation treatment, the serum potassium level normalized, but the symptoms of intestinal obstruction did not significantly improve. Therefore, we ruled out intestinal obstruction caused by inflammation and low potassium levels and identified the cause as mechanical intestinal obstruction.

Mechanical intestinal obstruction is often caused by adhesions, compression, or tumors. Previous reports have shown that appendicitis often results in internal hernias after adhesion to other parts of the abdominal cavity, leading to intestinal obstruction.^[7,8]^ After anti-infection treatment, the patient’s obstructive symptoms did not improve, and enhanced CT revealed clear obstructive points. The patient’s clinical symptoms were consistent with manifestations of mechanical intestinal obstruction, and surgical treatment was performed. Intraoperatively, it was confirmed that there was an obstruction in the ileocecal region and that the terminal ileum was compressed by a fibrotic band. This type of mechanical small-intestinal obstruction caused by an appendiceal abscess is relatively rare in clinical practice. To our knowledge, this is among the few reported cases in which an appendiceal abscess caused mechanical obstruction at the ileocecal region, as most cases occur outside the terminal ileum.^[9,10]^ We speculated that localized inflammatory infiltration and abscess formation led to the formation of fibrotic bands compressing the terminal ileum. This differs from the more common paralytic ileus due to peritonitis, highlighting the importance of considering mechanical etiologies for persistent obstruction. However, we are not sure whether this fibrotic band (cord) is congenital, caused by appendicitis, or caused by chronic inflammation. Although appendiceal abscess-associated MSBO is rare, accumulating evidence across clinical nutrition, systemic inflammatory diseases, and surgical outcome research highlights that sustained inflammatory burden and metabolic stress can drive tissue dysfunction and worsen outcomes when escalation of care is delayed.^[11–13]^ These findings support a strategy of close monitoring and timely surgical decision-making when conservative management fails.

Regarding the choice of surgical approach, our first step was to exclude tumor lesions. Laparoscopic exploration revealed severe swelling and adhesions in the ileocecal area, which was converted to open exploration. Intraoperative biopsy and rapid frozen pathology suggest the presence of inflammatory lesions (excluding tumors).^[14]^ Considering that abscess drainage alone cannot relieve mechanical compression at the end of the ileum and that the inflammatory infiltration of the diseased intestinal tract is severe, 1-stage resection can avoid the risk of intestinal fistula. Finally, ileocecal resection, followed by ileocolonic anastomosis, was performed. Before anastomosis, we confirmed that the blood supply and tension of the proximal and distal intestinal tracts were good, ensuring smooth postoperative recovery.

In conclusion, this is a complex case of adult appendiceal abscess combined with mechanical small-intestinal obstruction. The diagnosis and treatment process involves differentiation between inflammatory and mechanical intestinal obstruction, selection of surgical timing, and development of personalized treatment strategies. It is necessary to break through the mindset of “conventional conservative treatment.” When patients experience progressive obstructive symptoms, conservative treatment is ineffective, and infection indicators continue to increase; therefore, active surgical intervention should be performed. During surgery, a comprehensive evaluation of the cause of obstruction (especially excluding tumors) is needed, and a reasonable surgical procedure should be selected based on the degree of local inflammation.

## 4. Conclusion

This case underscores that when symptoms of obstruction do not resolve with conservative management of appendiceal abscess, mechanical obstruction should be suspected, and early surgical exploration may be lifesaving.

## Acknowledgments

The authors would like to thank the patient for his contribution to this research. The authors also thank Editage (www.editage.cn) for its linguistic assistance during the preparation of this manuscript.

## Author contributions

**Conceptualization:** Guangwei Gong.

**Data curation:** Yang Li, Jiaqing Lin, Wei Zhu.

**Investigation:** Kun Yang, Zhaopu Li, Jiaqing Lin, Tao Xu.

**Methodology:** Kun Yang, Guangwei Gong.

**Resources:** Zhaopu Li, Yang Li, Jiaqing Lin, Tao Xu, Wei Zhu.

**Visualization:** Zhaopu Li, Tao Xu.

**Formal analysis:** Yang Li, Guangwei Gong.

**Software:** Wei Zhu.

**Supervision:** Guangwei Gong.

**Writing – original draft:** Kun Yang.

**Writing – review & editing:** Kun Yang.
